# Immunomodulatory Effects of the Pea Defensin Psd1 in the Caco-2/Immune Cells Co-Culture upon *Candida albicans* Infection

**DOI:** 10.3390/ijms24097712

**Published:** 2023-04-23

**Authors:** Ivan V. Bogdanov, Serafima I. Fateeva, Alexander D. Voropaev, Tatiana V. Ovchinnikova, Ekaterina I. Finkina

**Affiliations:** 1 M.M. Shemyakin & Yu.A. Ovchinnikov Institute of Bioorganic Chemistry, Russian Academy of Sciences, 117997 Moscow, Russia; 2 G.N. Gabrichevsky Research Institute for Epidemiology and Microbiology, Admiral Makarov St. 10, 125212 Moscow, Russia

**Keywords:** pea defensin, Psd1, *Candida albicans*, candidiasis, immunomodulatory action, cytokines, epithelial immunity, xMAP

## Abstract

Candidiasis is one of the most common fungal diseases that can pose a threat to life in immunodeficient individuals, particularly in its disseminated form. Not only fungal invasion but also fatal infection-related inflammation are common causes of systemic candidiasis. In this study, we investigated in vitro immunomodulatory properties of the antifungal pea defensin Psd1 upon *Candida albicans* infection. Using the real-time PCR, we showed that Psd1 inhibited the antimicrobial peptide HBD-2 and pro-inflammatory cytokines IL-1 and IL-8 downregulation at mRNA level in epithelium cells caused by *C. albicans* infection. By using the Caco-2/immune cells co-culture upon *C. albicans* infection and the multiplex xMAP assay, we demonstrated that this pathogenic fungus induced a pronounced host defense response; however, the cytokine responses were different in the presence of dendritic cells or monocytes. We revealed that Psd1 at a low concentration (2 µM) had a pronounced immunomodulatory effect on the Caco-2/immune cells co-culture upon fungal infection. Thus, we hypothesized that the pea defensin Psd1 might be an effective agent in the treatment of candidiasis not only due to its antifungal activity, but also owing to its ability to modulate a protective immune response upon infection.

## 1. Introduction

*Candida albicans* is a common member of the mucosal microflora, which, however, causes such life-threatening bloodstream infections as candidemia and disseminated candidiasis in immunocompromised patients and in patients receiving immunosuppressive therapy [1]. It is generally accepted that the high prevalence of candidiasis in such patients is associated with a decrease in immune defense. However, some data show that the natural selection of more virulent strains also takes place in this case [2].

*C. albicans* is a dimorphic fungus, growing in yeast and hyphal forms. The morphological plasticity of *C. albicans* is an important virulence determinant, since fungal hyphae penetrate epithelial and endothelial barriers, causing systemic infections [3]. The intestinal mucosal barrier plays an important role in protection against an invasion of *C. albicans*; however, intestinal flora disorder, increased epithelium permeability due to inflammatory diseases, and impaired host immune responses increase the risk of invasive fungal infections [1,3,4].

After interaction with epithelium *C. albicans* cell adhesion and invasion via induced endocytosis and active penetration of fungal hyphae take place [5]. Epithelial activation by *C. albicans* induces pro-inflammatory immune responses (e.g., IL-8/CXCL8), which results in the recruitment of innate immune cells, particularly neutrophils that are, in turn, activated by GM-CSF, G-CSF, and IL-1 family members [3]. Neutrophils protect directly through phagocytosis and neutrophil extracellular trap (NET) formation and indirectly via immunological crosstalk with epithelial TLR4. MIP-3α/CCL20 and human β-defensin 2 (HBD-2) secretion recruits mucosal-homing CCR6-expressing dendritic cells, which will process fungal antigens and activate Th immunity, including Th-17 cells. Th-17 cells secrete interleukins IL-17A and IL-17F, which stimulate a variety of cells (e.g., epithelial cells and fibroblasts) to produce antimicrobial peptides (AMPs), metalloproteases, and chemokines, which promotes neutrophil recruitment and activation, ultimately resulting in fungal clearance. Th-17 cells also secrete IL-22, which limits fungal growth and maintains epithelial barrier function [3]. Inflammatory cytokines IL-12 and IL-23 have also been shown to play a pivotal role in anti-Candida immunity [6]. At the same, uncontrolled inflammation can lead to tissue damage and progression of the infection [7].

Multi-resistant strains of *C. albicans* are becoming more common and show reduced susceptibility to conventional antifungal drugs, including triazoles, polyenes, and echinocandins. Therefore, the need to search for new molecules that target these pathogenic species is now as urgent as ever. In addition, recent data show that a combination of antifungal and immunomodulatory therapy may be more effective for the treatment of high-mortality systemic mycoses [8].

Host defense AMPs playing an important role in innate immunity are considered nowadays as promising candidate substances for the development of new drugs [7,9]. It is well known that mammalian AMPs do not only possess antimicrobial activity, but also have immunomodulatory action on various immune cells. The most characterized examples of such AMPs are cathelicidin LL37 and human defensins [7]. Human defensins play a complex role in host defense, exhibiting not only antimicrobial activity, but also controlling the infection through modulating various immune activities including chemotaxis, phagocytosis, and cytokine induction. These cationic peptides can both induce and suppress inflammatory responses by acting on specific immune cells directly or in complex with proteins, nucleic acids, and carbohydrates. Human defensins implement their immunomodulatory action through a wide range of immune receptors including different TLRs and chemokine receptors [10].

Defensins consist of a conservative class of AMPs that have been found in animals, plants, fungi, and bacteria. The latest data show that plant defensins do not only exhibit antimicrobial activity against human pathogenic microorganisms, but also possess immunomodulatory action. For instance, defensin from *Capsicum chinense* modulates the innate immune response of bovine mammary epithelial cells infected with *Staphylococcus aureus* by inducing the production of both pro-inflammatory cytokines TNF-α and IL-1β and anti-inflammatory IL-10 [11]. Another example is the engineered analog of plant defensins EgK5, which suppresses antigen-triggered proliferation of effector memory T-cells (T_EM_), a subset enriched among pathogenic autoreactive T-cells in autoimmune diseases [12]. Lentil defensin Lc-def, as we showed recently, inhibits the growth of different *Candida* species, as well as possesses immunomodulatory activity and increases the production of such essential for immunity to candidiasis pro-inflammatory cytokines as IL-12 and IL-17 by human immune cells [13].

The aim of this study was to investigate for the first time the immunomodulatory action of plant defensins upon the *Candida albicans* infection using the antifungal pea defensin Psd1 as a case study. The pea defensin Psd1 was chosen for these experiments due to a number of reasons. It exhibits pronounced antifungal activity and effectively inhibits the growth of different strains of *C. albicans* [14,15]. Psd1 interacts with specific cell wall/membrane lipid targets, such as glucosylceramide (GlcCer) and ergosterol, which determines its antifungal and antitumor action. At the same time, this peptide has low affinity with cholesterol-rich membranes, explaining the reduced toxicity of Psd1 to human cells [16,17]. Moreover, as demonstrated, Psd1 does not only inhibit the formation of lung metastasis nodules in a murine model in vivo, but also decreases the number of inflammatory cells in lung tissues [17]. We supposed that the latter can be determined by an immunomodulatory action of Psd1. In the present work, we simulated in vitro the development of intestinal candidiasis by using not only Caco-2 cells, but also Caco-2/immune cells co-culture upon the *C. albicans* infection that takes into account epithelial-immune cells crosstalk. By using real-time PCR and multiplex xMAP approaches we investigated the impact of the pea defensin Psd1 on epithelial and epithelial-immune cell responses upon fungal infection with *C. albicans*.

## 2. Results and Discussion

### 2.1. Influence of the Pea Psd1 on Defense Response of Intestinal-like Epithelial Cells upon the Candida albicans Infection

In the first stage of our work, we investigated the influence of pea defensin Psd1 on epithelial monolayer upon the *Candida albicans* infection. It is known that colonization of yeast cells on the epithelium surface elicits a protective immune response. This response includes upregulation and secretion of such AMPs as human beta defensins (HBD), cathelicidin LL37, and histatin. Infection-mediated activation of epithelial cells results in the production of such pro-inflammatory cytokines as IL-1β, IL-6, and IL-8/CXCL8. This causes recruiting of granulocytes and immune cells to the site of inflammation and the differentiation of CD4^+^ T-cells into Th-17 type, producing IL-17 and Th-1 type, actively producing IFNγ, which determines anti-*C*andida immunity. However, the formation of fungal hyphae on the epithelial cells leads to inhibition of AMPs secretion, a decrease in the production of pro-inflammatory cytokines, and eventually switching the immune response to Th-2 or T-reg types, characterized by the production of anti-inflammatory cytokines, which determines the development of systemic candidiasis [3,5].

**Figure 1:**
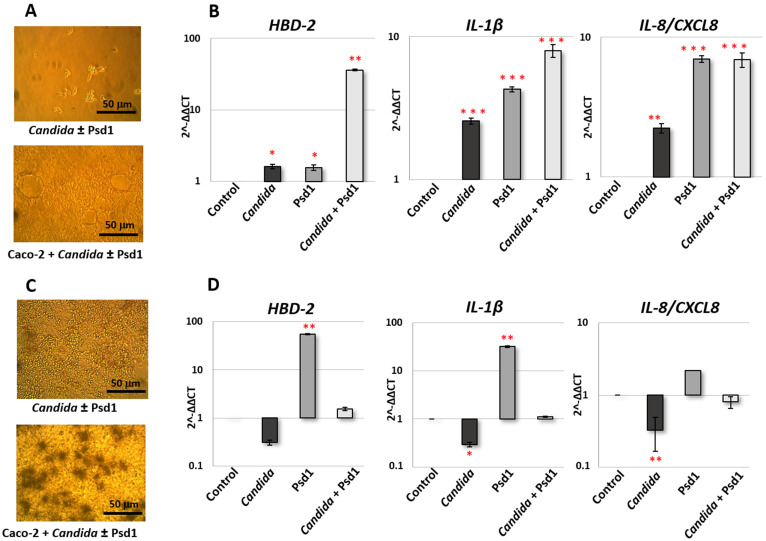
Caco-2 co-culture with *Candida albicans* in the presence or absence of 2 μM Psd1 after 4 (**A**) and 24 h (**C**) incubation (×400 magnification). Relative to control (untreated Caco-2 cells) expression of *HBD-2*, *IL-1β*, and *IL-8/CXCL8* genes by epithelial cells after 4 (**B**) and 24 h (**D**) incubation with *C. albicans* in the presence or absence of 2 μM Psd1. Data are representative of two different experiments ± SD (standard deviation). Significance levels are (relative to control): * *p* < 0.05, ** *p* < 0.01, *** *p* < 0.005.

Therefore, using real-time PCR we investigated the influence of the pea antifungal defensin Psd1 on the expression of *IL-1β*, *IL-8/CXCL8*, and *HBD-2* genes by epithelium cells upon the *Candida albicans* infection after 4 h and 24 h. For that polarized Caco-2 monolayer co-culture with *C. albicans* was used. The human epithelial cell line Caco-2 is originally derived from a colon carcinoma, but has the ability to spontaneously differentiate into a monolayer of cells with many properties typical of absorptive enterocytes of small intestine [18]. *C. albicans* v47a3 was used at the concentration of 5 × 10^4^ cells/mL (1.3 × 10^4^ cells/cm^2^) that did not disrupt epithelial monolayer after yeast cell growth for 24 h. Psd1 was active against this strain of *C. albicans* and exhibited 50% growth inhibition of yeast cells at a concentration of 25 μM and did not affect the morphology of the fungus (Supporting Materials, Figure S1). For investigation of its immunomodulatory effect, peptide was added to Caco-2/*C. albicans* co-culture at a final concentration of 2 μM, in which the pea defensin did not inhibit the growth of *C. albicans* (Figure 1A,C). We have previously shown that Psd1 was stable to proteolysis by *Candida albicans* enzymes [13]. Therefore, as we assume, the peptide could remain intact for a long time in Caco-2 monolayer co-culture with *C. albicans*.

Microscopic analysis revealed the absence of significant yeast growth after 4 h (Figure 1A). At the same time, an increase in the yeast cell concentration was observed after 24 h, and switching of *C. albicans* growth to the hyphal form in the presence of intestinal-like epithelial cells took place (Figure 1C). Analysis of gene expression after Caco-2 treatment during 4 h revealed that the presence of *C. albicans* cells, as expected, induced the production of HBD-2 and both pro-inflammatory cytokines by epithelial cells. The presence of Psd1 also upregulated all three genes. At the same time, a more pronounced activation of the protective immune response was observed in the presence of both pathogenic fungus and pea defensin, especially in the case of *HBD-2* gene (Figure 1B). Otherwise, gene expression of *HBD-2* and *IL-1β* and *IL-8*/*CXCL8* was downregulated after 24 h of infection by *C. albicans*. Immunostimulatory action of Psd1 was observable 24 h after the infection. It is important to note that the presence of the pea defensin significantly reduced the inhibitory effect of pathogenic fungus on the production of pro-inflammatory cytokines and HBD-2 (the difference between *Candida* and *Candida* with Psd1 is significant for all three genes, *p* < 0.05) (Figure 1D).

Thus, we concluded that Psd1 possesses immunomodulatory action on intestinal-like epithelial cells and may reduce pathogen-induced suppression of the protective epithelial response upon the *C. albicans* infection.

### 2.2. Transport of the Pea Defensin Psd1 across the Intestinal Epithelial Barrier and Cytotoxicity Assay

**Figure 2:**
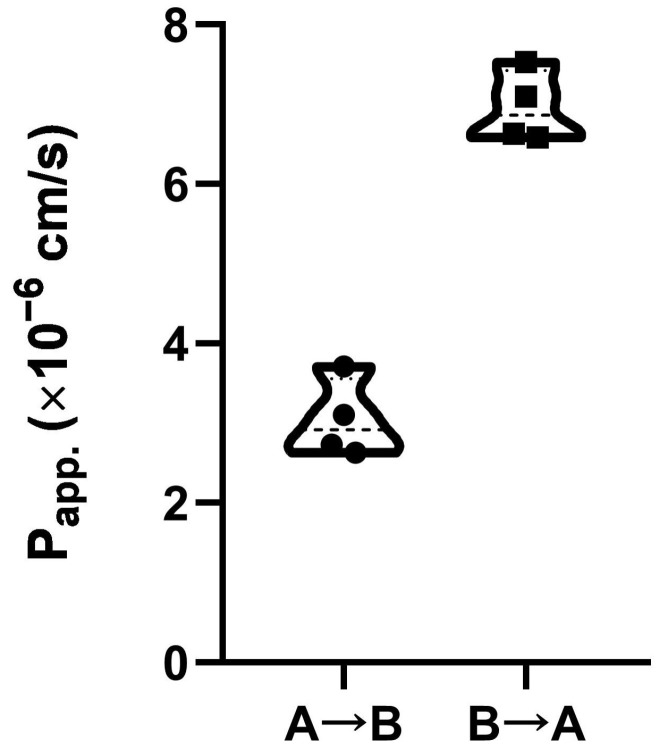
Bidirectional “apical-to-basolateral” (A→B) and “basolateral-to-apical” (B→A) transport of the pea defensin Psd1 across the Caco-2 epithelial barrier.

Next, we decided to investigate whether the pea defensin can pass through Caco-2 monolayer mimicking the gastrointestinal epithelial barrier. Proteins labeled with fluorescent probes are widely used for an assessment of the permeability through Caco-2 polarized monolayers [19]. Therefore, FITC-labelled Psd1 was obtained and used for the investigation of the intestinal absorption and efflux of the pea defensin in vitro. Estimation of “apical-to-basolateral” (A→B, absorptive) and “basolateral-to-apical” (B→A, secretory) bidirectional transport of the defensin across the Caco-2 epithelial barrier was performed. Apparent permeability A→B coefficients (P_app_) for Psd1 measured in four independent inserts were within the range of 2.63–3.71 × 10^−6^ cm/s (Figure 2), which predicts a moderate transepithelial absorption of the Psd1 in the human gut. The established relationship between the in vivo absorption of drugs in humans and P_app_ values allows to correlate P_app_ values ~2–10 × 10^−6^ cm/s with a 20–70% absorption in the gut which could be expected in humans [20]. Higher P_app_ in the B→A direction was observed: 6.96 ± 0.22 for B→A versus 3.04 ± 0.24 for A→B. The uptake (UR) and efflux ratios (ER) have also been calculated and were shown to be 0.44 and 2.29, respectively. An efflux ratio >2 is considered to indicate an active efflux [21]. Several efflux pumps have been shown to be present in Caco-2 cells, such as P-glycoprotein (ABCB1), breast cancer resistance protein (BCRP/ABCG2), or multi-drug resistance protein 2 (MRP2/ABCC2). The efflux ratio (ER) of 2.29 suggests that the pea defensin Psd1 may be subject to active efflux in the human intestine. However, this efflux is quite weak (ER < 3), as known model substrates of efflux pumps, such as vinblastine (substrate of MRP2), mitoxantrone (substrate of BCRP), and digoxin (substrate of P-gp), have ER > 10.

It was shown previously that pea Psd1 is not toxic for Beas-2B human bronchial epithelial cells [16]. Here we investigated the cytotoxic action of the pea Psd1 on human immune cells by using a human peripheral blood mononuclear cells (PBMCs) fraction. As expected, defensin had no cytotoxic activity on PBMCs. Cell viability of 100% was observed even at the peptide concentration of 50 μM, unlike membrane-active peptide melittin, which induced 50% cell death at a concentration of 6.25 μM (Supporting Materials, Figure S2).

Thus, we showed that the pea defensin Psd1 may be adsorbed by the intestinal-like epithelial cells and has no cytotoxic action on immune cells.

### 2.3. Direct Immunomodulatory Action of the Pea Defensin Psd1 on Human Dendritic Cells and Blood Monocytes

Further, we studied the direct immunomodulatory action of the pea defensin Psd1 on human immune cells. In the current work, we focused on the responses of such two key types of innate immune system cells upon the *Candida albicans* infection as monocyte-derived dendritic cells (moDCs) and monocytes. DCs patrol the peripheral tissues beneath mucosal surfaces and are recruited to the site of infection in response to secreted by epithelial cells chemokines and AMPs, particularly HBD, upon the microbial infection. Moreover, DCs play an important role in the initiation of adaptive T-cell-mediated immune responses against *C. albicans* [3,6]. Circulating monocytes are believed to play also an important role in immune responses to *Candida* infection, as they are recruited to the infected tissue where they differentiate into inflammatory macrophages [22]. Quantitative assessment of cytokines, chemokines, and growth factors in culture media at a protein level was performed by multiplex xMAP technology, which is based on the simultaneous usage of different types of magnetic microspheres conjugated with primary antibodies. Thus, the principle of multiplex xMAP technology is the same as in ELISA assay except that primary antibodies are conjugated to magnetic microspheres but not coated on polystyrene surfaces.

**Figure 3:**
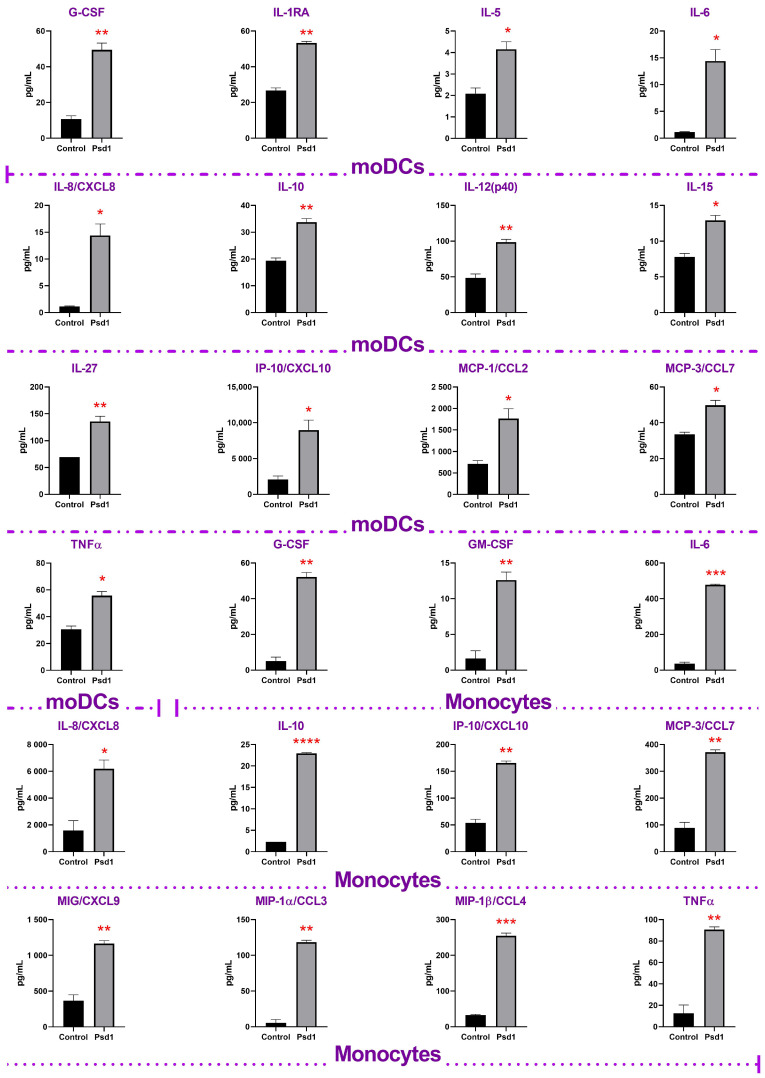
Profiles of cytokines, chemokines, and growth factors production by dendritic cells and monocytes in vitro in response to stimulation with 2 µM of Psd1. Error bars represent a standard deviation (±SD) between two replications. Significance levels are: * *p* < 0.05, ** *p* < 0.01, *** *p* < 0.0005, **** *p* < 0.0001.

In these experiments, human immune cells were treated for 24 h by Psd1 also at a concentration of 2 μM. We found that Psd1 activated immune cells and increased the production of pro-inflammatory (TNFα, IL-8, IL-12, IL-15, IP-10, MCP-1, MCP-3, MIG, MIP-1α, MIP-1β, GM-CSF), anti-inflammatory (IL-5, IL-10, G-CSF, IL-1RA) cytokines and chemokines, as well as cytokines with ambiguous action (IL-6, IL-27). It is interesting to note that the pea defensin increased almost the same analytes in both cultures, moDCs, and monocytes (Figure 3): TNFα, IL-6, IL-8, IP-10, MCP-3, and G-CSF. Moreover, the increase in several cytokines, such as IL-12 (*p* = 0.0087), IL-15 (*p* = 0.013), IL-27 (*p* = 0.011), and chemokine MCP-1 (*p* = 0.024) in response to incubation with Psd1 was statistically significant in moDCs, but failed to reach statistical significance in case of monocytes (Supporting Materials, Table S1).

As shown various cytokines produced by epithelial and immune cells play an important role in immunity upon the *Candida albicans* infection. Cytokines are involved not only in the recruiting of immune cells to the site of infection but also in immune cell communication, providing regulation of inflammatory response, and interaction between different components of innate and T-helper (Th) immunity [23]. A key role of not only pro-inflammatory cytokines TNFα, IL-6, IL-12, and IL-17, but also anti-inflammatory IL-4 and IL-10 has been established by using experimental models of *Candida albicans* infections and cytokine-deficient mice [23,24]. In our study we found that the pea defensin Psd1 increased production of TNFα which attracts and activates neutrophils for antifungal effector functions as well as IL-12, IL-10, and IL-6, playing a crucial role in Th1-mediated protective anti-Candida immune response.

Thus, we showed that the pea defensin has an immunomodulatory effect, and after transport through intestinal epithelium, it can stimulate immune cells and induce the production of various pro- and anti-inflammatory cytokines by various immune cells.

### 2.4. Role of Epithelial-Immune Cells Crosstalk in the Caco-2/Immune Cells Co-Culture upon the Candida albicans Infection

Model systems of cell co-culture are often used to study the mechanisms of candidiasis. On the one hand, Caco-2 cells co-culture with the *C. albicans* model is widely used for the study of intestinal infection [25]. On the other hand, immune cells co-culture with the *C. albicans* model is a common approach to study immune responses during fungal infection [26]. To the best of our knowledge, in this study for the first time we used Caco-2/immune cells co-culture with *C. albicans*, taking into account the communication between epithelial and immune cells upon the fungal infection.

The co-culture model consisted of immune cells (moDCs or monocytes) lying on the bottom of the culture well, and a Caco-2 polarized monolayer, mimicking intestinal epithelium, on a permeable insert. Immune cells were located on the serosal side of the epithelial barrier. In some inserts *Candida albicans* was loaded to the apical side of the epithelium, mimicking intestinal lumen colonization with this pathogenic fungus. This co-culture model allows yeast cells to contact only with the apical side of epithelial cells, but not with immune cells. Culture medium, containing cytokines, chemokines, and growth factors, produced by both epithelial and immune cells, was taken from basolateral chambers and analyzed by multiplex xMAP technology after incubation during 24 h when *C. albicans* cells switched to hyphal form.

To estimate the impact of *Candida albicans* on cytokine production by intestinal-like epithelium, Caco-2 polarized monolayer without immune cells was used. We did not find a pronounced immune response according to the production of cytokines, chemokines, and growth factors, including in our multiplex panel, by epithelial cells in response to infection with *C. albicans* for 24 h (Supporting Materials, Table S1). It is worth mentioning that the production of pro-inflammatory IL-8/CXCL8 by Caco-2 cells was downregulated by *Candida albicans*, which was also shown by us on the mRNA level in real-time PCR experiments (Figure 1). A decrease in concentrations of IL-13 and IL-18 was also observed.

**Figure 4:**
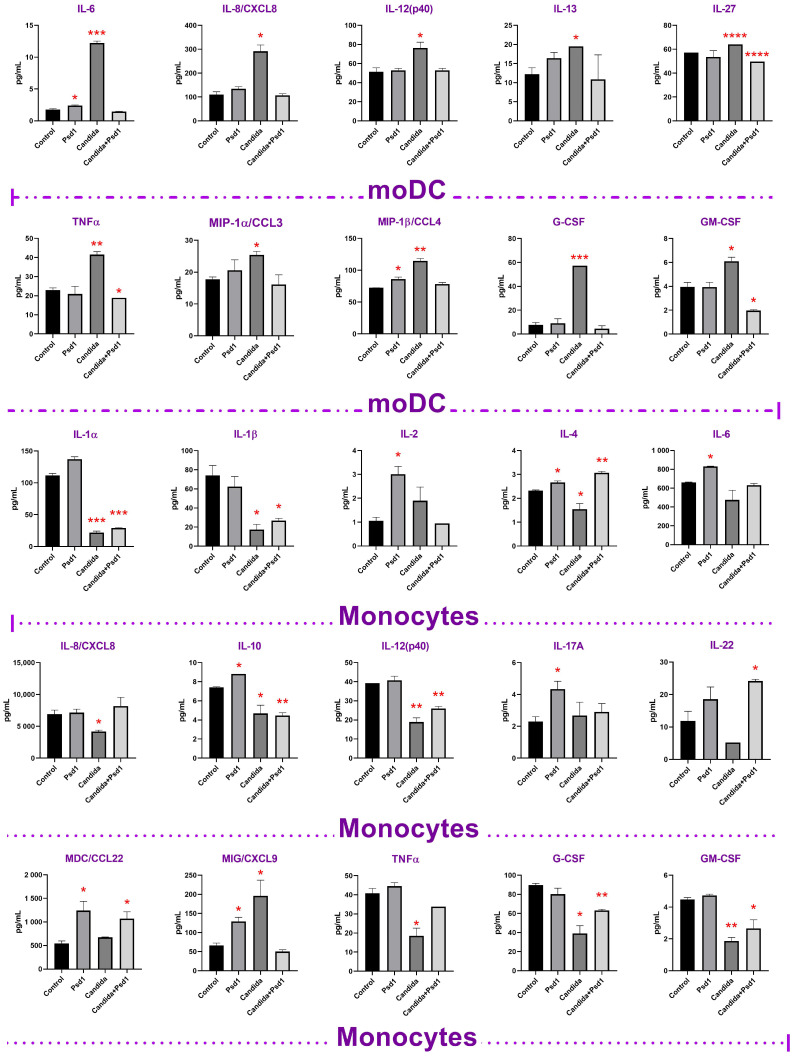
Profiles of cytokines, chemokines, and growth factors production by dendritic cells and monocytes in Caco-2/immune cells co-culture model. Error bars represent a standard deviation (±SD) between two replications. Significance levels are: * *p* < 0.05, ** *p* < 0.01, *** *p* < 0.0005, **** *p* < 0.0001.

**Figure 5:**
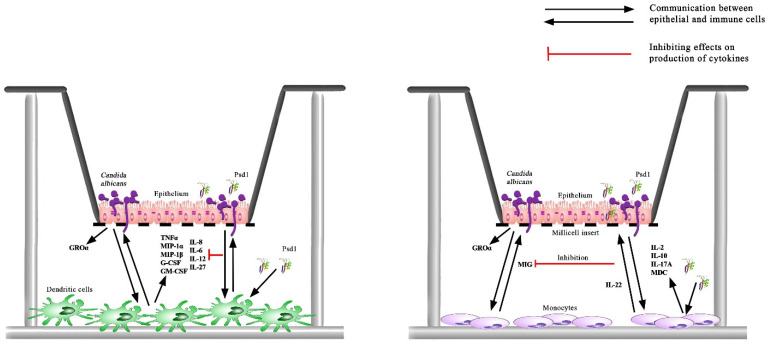
Cell communication in the Caco-2/immune cells co-culture upon the *Candida albicans* infection and immunomodulatory effect of the pea defensin Psd1 in this model system.

The situation was changed in the Caco-2/immune cells co-culture used. Interestingly, the immune response in the presence of dendritic cells and monocytes, which perform different immune functions and appear in the site of inflammation during infection at various time, was completely different. In Caco-2/dendritic cells co-culture an infection of epithelial cells by *C. albicans* induced production of many immune factors including cytokines TNFα, IL-6 and IL-12, chemokine IL-8/CXCL8, hematopoietic growth factors G-CSF and GM-CSF critical for polymorphonuclear neutrophils recruitment and activation [27], and MIP-1α and MIP-1β, taking part in recruiting of monocytes to the site of inflammation [26] (Figure 4; Supporting Materials, Table S1). It seems that tissue-resident DCs might be able to stimulate TNFα-mediated granulopoiesis, induce IL-8/CXCL8-mediated chemotaxis of neutrophils to the site of infection, recruit and activate neutrophils by G-CSF and GM-CSF production, and recruit monocytes by upregulated MIP (Figure 5). We also found that infection of epithelial cells in Caco-2/DCs co-culture by *C. albicans* induced slight, but statistically significant (*p* < 0.0001), production of IL-27, which is a member of the IL-12 family of cytokines and is produced by myeloid cells in response to selected *Candida* spp. (Figure 5) [28].

Quite a different situation was observed in the case of Caco-2/monocytes co-culture, where significant inhibition in the production of many cytokines, chemokines, and growth factors studied was registered (Figure 4 and Figure 5; Supporting Materials, Table S1). Possibly, infiltrated monocytes might be attracted by MIP-1 produced by DCs during later stages of fungal infection and do not exhibit pro-inflammatory properties, but produce MIG/CXCL9 chemokine which plays an important role in the recruitment of activated T-cells to the site of infection. MIG/CXCL9 enhances both Th-1 and Th-2 polarization, but they selectively attract Th-1 cells while inhibiting the migration of Th-2 cells [29].

Thus, by using Caco-2/immune cells co-culture we showed an important role of communication between epithelial and immune cells upon the *C. albicans* infection, which leads to pronounced activation of the immune response. Our data demonstrated that using epithelial cells in co-culture with various types of immune cells may provide new data regarding cellular response to fungal infection.

### 2.5. Impact of the Pea Defensin Psd1 on Cellular Response in the Caco-2/Immune Cells Co-Culture upon the Candida albicans Infection

In the next stage of our work, we investigated the influence of pea defensin Psd1 on cellular response in Caco-2/immune cells co-culture upon the *Candida albicans* infection. For that Psd1 at the concentration of 2 μM was added to the apical side of the epithelium in the presence or absence of pathogenic fungal cells. Analysis of immune factors production was performed also after incubation for 24 h when the hyphal form of *C. albicans* on the surface of the epithelium was observed.

We revealed that Psd1 itself had no significant direct immunomodulatory action on epithelial cells. Only the increase in IL-6, IL-8/CXCL8, and IP-10 was observed. Upregulation of IL-8/CXCL8 in Caco-2 cells treated by Psd1 was also shown in real-time PCR experiments (Figure 1 and Figure 4; Supporting Materials, Table S1). Surprisingly, there was also no significant induction of an immune response in Caco-2/immune cells co-culture with Psd1, in contrast to the direct stimulation of dendritic cells or monocytes by the peptide (Figure 3 and Figure 4; Supporting Materials, Table S1). A slight decrease in the production of pro-inflammatory cytokine IL-1β, IL-6, and MIG/CXCL9 chemokine was observed in Caco-2/dendritic cells co-culture. On the contrary, in the case of Caco-2/monocytes co-culture, we revealed a statistically significant increase in the production of IL-2, IL-10, MDC as well as IL-17, which is mainly produced as known by T helper 17 cells (Th17), recruits and activates neutrophils, and plays a key role in immune defense against *Candida albicans* infection (Figure 4 and Figure 5; Supporting Materials, Table S1) [6]. It is worth noting that the direct action of Psd1 on monocytes upregulated the same cytokines/chemokines (Figure 3; Supporting Materials, Table S1). As a result, we concluded that the range of soluble immune factors in the Psd1-treated Caco-2/immune cells co-culture is mainly determined by crosstalk between epithelial and immune cells and, to a lesser extent, defensin penetration through the epithelial barrier and subsequent direct stimulation of immune cells.

Further, we found that the pea defensin changed immune response and mainly negated the stimulatory or inhibitory effects of the *Candida albicans* infection on co-cultures of Caco-2 with dendritic cells or monocytes, respectively. In particular, the presence of Psd1 inhibited the production of TNFα, IL-6, IL-8/CXCL8, IL-12, IL-27, G-CSF, GM-CSF, and MIPs, induced by *C. albicans* in Caco-2/dendritic cells co-culture, although defensin itself had a stimulating effect on dendritic cells (Figure 3, Figure 4 and Figure 5; Supporting Materials, Table S1). Unlike, the presence of Psd1 decreased inhibiting action of pathogenic fungus on the production of IL-8/CXCL8, IL-12, TNFα, G-CSF, and GM-CSF in Caco-2/monocytes co-culture. Moreover, Psd1 in this system increased the production of IL-4, taking part not only in anti-inflammatory Th-2 type immune response, but also, as proposed, in the induction of CD4^+^ Th-1 protective responses due to combined action on the cells of innate and adaptive immunity [30]. Moreover, Psd1 statistically significantly (*p* < 0.05) increased in Caco-2/monocytes co-culture upon the *C. albicans* infection the production of IL-22, which is mainly produced by Th-17 cells, controls the growth of yeasts and contributes to the host’s epithelial integrity in the absence of acquired Th-1 type immunity [31] (Figure 4 and Figure 5; Supporting Materials, Table S1). Our data suggested that not only adaptive Th-17 cells, but also some innate antigen-presenting cells such as dendritic cells and monocytes, might act as a source of IL-17 and IL-22 during infection by *Candida albicans*. On another hand, Psd1 particularly did not affect reduced by *C. albicans* production of IL-1, IL-10, and IL-12, but decreased the production of MIG/CXCL9 chemokine in Caco-2/monocytes co-culture upon the fungal infection (Figure 4 and Figure 5; Supporting Materials, Table S1).

Thus, we showed that the pea defensin Psd1 poorly affected the cytokine profiles of co-cultured epithelial and immune cells, but had a pronounced immunomodulatory action on these co-culture models upon the *C. albicans* infection, modulating the mechanism of cellular response. We assumed that Psd1 may prove to be effective during the development of candidiasis by prevention of fungal-mediated suppression of the host defense response of epithelial cells and is able to remodulate excessive pro-inflammatory response induced by immune cells. At the same time, it cannot be ruled out that the observed effects of Psd1 upon the infection may also be due to the influence of the pea defensin on the metabolism and pathogenicity of *C. albicans*.

Summing up all data obtained, we showed that the pea defensin Psd1 does not only have anti-Candida activity, but also exhibits immunomodulatory action on epithelial immunity during fungal infection. However, whether this immunomodulatory action will help the immune system to cope with the infection in vivo or not is to be verified in experiments with mice models of intestinal candidiasis.

## 3. Materials and Methods

### 3.1. Materials

The clinical isolate of *Candida albicans* (v47a3) was collected from the patient with human immunodeficiency virus (HIV) infection and mucosal candidiasis and provided by G.N. Gabrichevsky Research Institute for Epidemiology and Microbiology (Moscow, Russia). This strain was susceptible to conventional antifungal agents except for a slight increase in the minimal inhibitory concentrations for triazole derivatives as was shown using the broth dilution method according to [31] (Supporting Materials, Tables S2 and S3). Strain stock was kept at −70 °C in 10% non-fat milk with 10% glycerol. Melittin (>98% pure) synthesized using a standard solid-phase method was provided by Dr. Sergey V. Sychev in M.M. Shemyakin and Yu.A. Ovchinnikov Institute of Bioorganic Chemistry of the Russian Academy of Sciences (Moscow, Russia).

### 3.2. Recombinant Production and Characterisation of the Pea Defensin Psd1

The pea defensin from *Pisum sativum*, designated as Psd1 (UNIPROT P81929), was obtained as described previously [13]. The recombinant peptide was overexpressed in *Escherichia coli* and purified from clarified cell lysate using metal chelate chromatography, cleavage of the fusion protein His8-TrxL-Psd1 with cyanogen bromide and two-stage reversed-phase high-performance liquid chromatography (RP-HPLC). Homogeneity and the identity of the recombinant peptide sample were confirmed by SDS-PAGE, MALDI-TOF mass spectrometry, and CD spectroscopy. Purified recombinant Psd1 was free of lipopolysaccharide and other pyrogens, which was checked by comparing IL-1β levels in control and Psd1-containing wells with monocytes in case of direct stimulation with the peptide, as primary human monocytes represent a highly pyrogen-sensitive culture.

Antifungal activity of recombinant Psd1 against *C. albicans* v47a3 was tested by the microdilution method using 96-well microplates mainly as described for this defensin in [15]. Yeast cells in half-modified YPD broth at a final concentration of 10^4^ cells/mL were used. Final Psd1 concentrations in the wells were 100, 50, 25, 12.5, 6.25, 3.125, and 1.56 μM. Plates were incubated at 30 °C for 24 h. Yeast growth was estimated using an inverted microscope as well as by measuring the optical density at 540 nm. The percentage of growth inhibition was defined as growth inhibition(%) = ((A_control_ − A_sample_)/A_control_) × 100%. All of the experiments were performed twice in triplicate.

### 3.3. Human Cell Lines and Cultures

Primary peripheral blood mononuclear cells (PBMCs) collected from healthy donors were purchased from American Type Culture Collection (ATCC PCS-800-011). Primary CD14^+^ blood monocytes were isolated from PBMCs by adherence according to standard technique [32]. Monocyte-derived mature dendritic cells (moDCs) were obtained from CD14^+^ blood monocytes according to the previously reported protocol [33]. Briefly, blood monocytes were incubated in a complete RPMI-1640 medium (Capricorn Scientific, Ebsdorfergrund, Germany), containing 10% fetal bovine serum (FBS, Capricorn Scientific, Ebsdorfergrund, Germany), 1× antibiotic-antimycotic solution (Invitrogen, Waltham, MA, USA), 800 U/mL rhGM-CSF (Sci-Store, Moscow, Russia) and 500 U/mL rhIL-4 (Sci-Store) for 3 days in CellXpert C170i CO_2_-incubator (Eppendorf, Hamburg, Germany). Fresh complete RPMI-1640 medium with rhGM-CSF and rhIL-4 was added to the culture on day 3. On day 5, fresh complete RPMI-1640 containing 100 U/mL rhTNF-α (Sigma-Aldrich, St. Louis, MO, USA) as well as 800 U/mL rhGM-CSF and 500 U/mL rhIL-4 was added and the culture was incubated for another 4 days until full maturation.

Colorectal adenocarcinoma Caco-2 cell line (ATCC HTB-37) was cultured in complete DMEM/F12 (1:1) (Gibco, Waltham, MA, USA) medium containing 10% FBS (Capricorn Scientific) and 1× antibiotic-antimycotic solution (Invitrogen) in a humidified CO_2_-incubator (5% CO_2_, 37 °C).

For growing cells, mimicking epithelial barriers in vitro, Caco-2 cells were seeded onto 24-well polycarbonate Millicell cell culture inserts (0.4 μm, 0.6 cm^2^ surface area) (Millipore, Burlington, MA, USA), precoated with 0.2% bovine gelatin (Sigma-Aldrich), at a density of 2.5 × 10^5^ cells/cm^2^. The cells were grown for 21 days in a complete DMEM/F12 (1:1) medium with re-feeding every 2–3 days with a fresh complete medium. The integrity of the Caco-2 cell monolayer was checked by measuring the transepithelial electrical resistance (TEER) using a Millicell-ERS Voltohmmeter (Millipore). Only cell monolayers with TEER > 400 Ωcm^2^ (after subtracting TEER in blank inserts without Caco-2 cells) were used in transport across epithelial monolayer and cytokine production experiments.

*Candida albicans* cells in stock were inoculated onto modified YPD (yeast extract 5 g/L, peptone 10 g/L, glucose 10 g/L) agar plates, and incubated for 24 h at 37 °C. After replating, cells were cultured in complete RPMI-1640 (Capricorn Scientific) without antibiotics at 37 °C to optical density 1.0 at 540 nm, centrifuged, and diluted up to the concentration of 5 × 10^4^ cells/mL in fresh RPMI-1640 without FBS and antibiotics. In the case of studying combined action on the epithelium of yeast cells and the pea Psd1 recombinant peptide was dissolved in water at the concentration of 200 μM and was added to yeast cell suspension up to the final concentration of 2 μM. Yeast growth and morphology in the presence or absence of peptide under co-cultivation with Caco-2 cells in a 24-well plate were estimated using an inverted microscope, Olympus CKX41 (Olympus, Tokyo, Japan).

### 3.4. Labeling of the Pea Defensin Psd1 with FITC

The recombinant Psd1 was labeled with fluorescein isothiocyanate isomer I (FITC) (Sigma-Aldrich). For this, 0.5 mg of Psd1 was reconstituted in 20 µL of DMSO, then added to 100 µL of the buffer for coupling (0.1 M sodium carbonate, 0.1 M sodium bicarbonate, pH 10.8) and 3.7 mg of FITC in 100 µL of DMSO. The coupling reaction was conducted for 2 h at room temperature in the dark. In order to purify FITC-Psd1, the reaction mixture was loaded onto the PD10 gel-filtration column (GE Healthcare, Chicago, IL, USA) previously equilibrated with transport buffer (Hank’s balanced salt solution, containing 1 mM MgCl_2_, 1 mM CaCl_2_, and 10 mM D(+)glucose, pH 7.4).

### 3.5. Transport of FITC-Psd1 across the Caco-2 Epithelial Barrier

Transport of FITC-Psd1 across the Caco-2 epithelial barrier in vitro was performed in the transport buffer. Bidirectional “apical-to-basolateral” (A→B) and “basolateral-to-apical” (B→A) transport of FITC-Psd1 in the transport buffer across epithelial barrier was investigated. The “apical-to-basolateral” assay was initiated by adding 0.4 mL of 2 µM FITC-Psd1 to the apical (luminal) side of the monolayer and 0.7 mL of the transport buffer (pH 7.4) to the basolateral side of the monolayer. The “basolateral-to-apical” assay was performed in a similar manner, except that 0.4 mL of the transport buffer (pH 7.4) was added to the apical side and 0.7 mL of 2 µM FITC-Psd1 to the basolateral (serosal) side. All solutions were pre-warmed to 37 °C before taking into the transport experiment. Transport in vitro across Caco-2 barriers was conducted for 90 min in 4 independent inserts for each studied transport variant.

An apparent permeability coefficient (P_app_) was calculated for each insert according to the following equation:

P_app_ = (V/(A × Ci)) × ΔC/Δt, (1)

where V is the volume of the acceptor chamber, A is the area of the membrane insert, Ci is the initial concentration of Psd1, and ΔC/Δt is the solute flux across the barrier. Uptake ratio:

UR = P_app_(A→B)/P_app_(B→A), (2)

and efflux ratio:

ER = P_app_(B→A)/P_app_(A→B), (3)

for Psd1 were calculated from averaged apparent permeability coefficients measured in 4 independent inserts. Monolayer integrity was checked by measuring TEER before and after the end of the experiment.

### 3.6. Cytokines/Chemokines/Growth Factors Production by Human Cell Cultures

Monocytes and moDCs were seeded into the wells of 48- and 24-well plates in the complete RPMI-1640 medium supplemented with 10% human AB serum (HABS, Capricorn Scientific) without antibiotics 24 h prior to the experiment, and kept in a humidified CO_2_-incubator (5% CO_2_, 37 °C). After 24 h, the medium in the wells of the 48-well plate was replaced by fresh complete RPMI-1640 medium with 10% HABS, supplemented with 2 µM of Psd1 for the sample wells or fresh medium alone for the control wells. Medium in the wells of the 24-well plate was replaced by fresh complete RPMI-1640 medium with 10% human serum without antibiotics. Then, Millicell inserts with Caco-2 monolayers with TEER > 400 Ωcm^2^ were placed into the wells of the 24-well plate, containing monocytes or moDCs. Wells with Millicell inserts with Caco-2 monolayers without immune cells were used as control wells. Medium in apical chambers of Millicell inserts was replaced by fresh complete RPMI-1640 with 10% human serum medium containing Psd1 at the final concentration of 2 µM or *Candida albicans* at the concentration of 5 × 10^4^ cells/mL or yeast cells together with the pea defensin at the same concentration. Fresh complete RPMI-1640 medium only with 10% human serum and without antibiotics was used in control wells. Plates were kept in CO_2_-incubator (5% CO_2_, 37 °C) for another 24 h. Culture supernatants from the wells of the 48-well plate and basolateral chambers of the 24-well plate were collected and stored at −70 °C degrees less than one month prior to the analytes assessment.

### 3.7. Cytokines/Chemokines/Growth Factors Assessment by Multiplex-Based Assay

The following 48 analytes were measured at a protein level by multiplex xMAP technology using the MILLIPLEX Human Cytokine/Chemokine/Growth Factor Panel A kit (HCYTA-60K-PX48, Merck, Darmstadt, Germany): sCD40L, EGF, Eotaxin-1/CCL11, FGF-2/FGF-basic, Flt-3 ligand, Fractalkine/CX3CL1, G-CSF, GM-CSF, GROα/CXCL1, IFNα2, IFNγ, IL-1α, IL-1β, IL-1RA, IL-2, IL-3, IL-4, IL-5, IL-6, IL-7, IL-8/CXCL8, IL-9, IL-10, IL-12(p40), IL-12(p70), IL-13, IL-15, IL-17A/CTLA8, IL-17E/IL-25, IL-17F, IL-18, IL-22, IL-27, IP-10/CXCL10, MCP-1/CCL2, MCP-3/CCL7, M-CSF, MDC/CCL22, MIG/CXCL9, MIP-1α/CCL3, MIP-1β/CCL4, PDGF-AA, PDGF-AB/BB, RANTES/CCL5, TGFα, TNFα, TNFβ, and VEGF-A. Multiplex-based assay read-out was performed using the MAGPIX system (Merck) with the xPONENT 4.2 software (Merck) in accordance with the manufacturer’s instruction with overnight incubation of the samples with primary antibodies. A final analysis was carried out with the MILLIPLEX Analyst v5.1 software (Merck). Measurements were performed twice for each sample. Release of the analytes in control and experimental samples was compared with an unpaired two-sample *t*-test using GraphPad Prism v.8.0.1 (GraphPad Software, Inc., San Diego, CA, USA). The *p* values ≤ 0.05 were considered significant.

### 3.8. Gene Expression of Pro-Inflammatory Cytokines and Antimicrobial Peptides

#### 3.8.1. Stimulation of Caco-2 Cells

Caco-2 cells were seeded into the wells of 24-well plates in the complete RPMI-1640 medium containing 10% FBS and 1X antibiotic-antimycotic solution and kept in a humidified CO_2_-incubator (5% CO_2_, 37 °C). Cells were grown for 18 days and the medium was changed twice a week. The presence of a cell monolayer was checked using an inverted microscope and the plates were incubated one more week for complete polarization of Caco-2 cells. Caco-2 cells were stimulated by adding fresh complete RPMI-1640 medium without antibiotics, containing Psd1 at the final concentration of 2 µM or *Candida albicans* at the concentration of 5 × 10^4^ cells/mL (1.3 × 10^4^ cells per cm^2^ of Caco-2 monolayer) or yeast cells together with the pea defensin at the same concentration. RPMI-1640 medium only was used in control wells.

#### 3.8.2. RNA Isolation and cDNA Synthesis

Total RNA was isolated from Caco-2 cells using TRIzol reagent (Invitrogen) according to the manufacturer’s instructions after 4 or 24 h of stimulation and cell washing by RPMI-1640 medium. The quality and quantity of total RNA were determined by the A260/280 ratio using NanoPhotometer NP80 (Implen GmbH, München, Germany) as well as agarose gel electrophoresis (Supporting Materials, Figure S3). RNase inhibitor (Evrogen, Moscow, Russia) was added to the final preparation of total RNA. Two μg of total RNA was taken to synthesize the cDNA using MINT-Universal cDNA synthesis kit (Evrogen, Moscow, Russia) containing MMLV-based reverse transcriptase following the manufacturer’s instructions.

#### 3.8.3. Real-Time PCR

Real-time PCR was performed on CFX Opus 96 Real-Time PCR System (Bio-Rad, Hercules, CA, USA) using the SYBR Green PCR kit (Evrogen, Moscow, Russia) and target-specific primers [34,35] (Supporting Materials, Table S4). The internal control housekeeping gene GAPDH was amplified simultaneously in separate reaction tubes [36]. The reaction conditions were set as follows: initial heating at 95 °C for 3 min, followed by 50 cycles of reactions at 95 °C (15 s), followed by 62 °C (20 s), and finally 72 °C (30 s). Melt curves were obtained using a temperature increment from 50 to 95 °C at 0.5 °C/5 s (Supporting Materials, Figure S4). The threshold cycle number (CT) of reactions was determined using the CFX Maestro 2.2 (version 5.2.008.0222) software (Bio-Rad, Hercules, CA, USA). The efficiency of PCR was monitored by using LinRegPCR online tool (https://www.gear-genomics.com/rdml-tools/) [37]. The relative expression of the genes of interest was calculated by normalization to GAPDH using the standard 2^−ΔΔCT^ method. Unstimulated Caco-2 cells after 4 and 24 h of incubation were used as controls. Experiments were performed twice in six technical replications. Gene expression in controls and experimental samples was compared with a *t*-test using GraphPad Prism v.8.0.1. The *p*-values ≤ 0.05 were considered significant.

### 3.9. Cytotoxicity Assay

The cytotoxic properties of the pea defensin Psd1 were investigated by resazurin-based cell cytotoxicity assay as previously described [13]. In brief, PBMCs were seeded in 96-well plates at 2 × 10^6^ cells per well in RPMI-1640 supplemented with 10% FBS and kept in a CO_2_ incubator for 24 h. After this, serial two-fold dilutions of defensin in a complete culture medium were added to the wells at final concentrations from 0.2 to 50 μM and the plates were incubated for 24 h. Resazurin (Sigma) was added at a final concentration of 70 µM, and the plates were incubated overnight (16–18 h). Fluorescent resorufin was registered using a 535/595 filter at PlateReader AF2200 (Eppendorf). Untreated cells were used as negative controls. Non-ionogenic detergent Triton X-100 and membrane-active peptide melittin from honeybee venom were used as positive controls. The cell viability was calculated according to the following equation: cell viability (%) = (F_sample_/F_control_) × 100%. Cell lysis was monitored with an Olympus CKX41 microscope.

## 4. Conclusions

In the current study, we investigated in vitro host defense response upon the epithelium colonization by *Candida albicans* and the immunomodulatory effect of the pea defensin Psd1 upon the fungal infection. For this, we used not only Caco-2 cells co-culture with *C. albicans*, but also for the first time Caco-2/immune cells co-culture upon the fungal infection to take into account epithelial-immune cells crosstalk. We revealed that Psd1 was able to pass through Caco-2 cell monolayer and exhibited immunomodulatory effects on both epithelial and immune cells. We demonstrated that epithelial cells themselves responded to the *C. albicans* infection by producing pro-inflammatory cytokines and antimicrobial peptide HBD-2, but switching pathogenic fungus to hyphal form led to suppression of the immune response. At the same time, Psd1 reduced pathogen-induced suppression of the protective immune response of epithelial cells upon the fungal infection. We showed that *C. albicans* induced a pronounced host defense response in Caco-2/immune cells co-culture, but in the presence of dendritic cells or monocytes, which perform various functions during infection, the immune response realized differently. We revealed that Psd1 had a pronounced immunomodulatory effect on the Caco-2/immune cells co-culture upon the *C. albicans* infection, modulating the mechanism of the immune response. However, to conclude whether the pea defensin Psd1 might be a promising antifungal peptide with immunomodulatory action, further experiments in vivo with mice models of intestinal candidiasis should be performed.

## Data Availability

All data generated and analyzed during this study are included in this published article and its Supplementary Materials File.

## Supplementary Materials

The following supporting information can be downloaded at: https://www.mdpi.com/article/10.3390/ijms24097712/s1.
