# Supplementary material for: Immunomodulatory Effects of the Pea Defensin Psd1 in the Caco-2/Immune Cells Co-Culture upon Candida albicans Infection
Source: Int J Mol Sci. 2023 Apr 23;24(9):7712. doi: 10.3390/ijms24097712 (PMC10178127; doi:10.3390/ijms24097712)
Supplement: Supplementary file 1 [file ijms-24-07712-s001.zip › IJMS_Finkina_Supporting information.pdf]

Supporting information

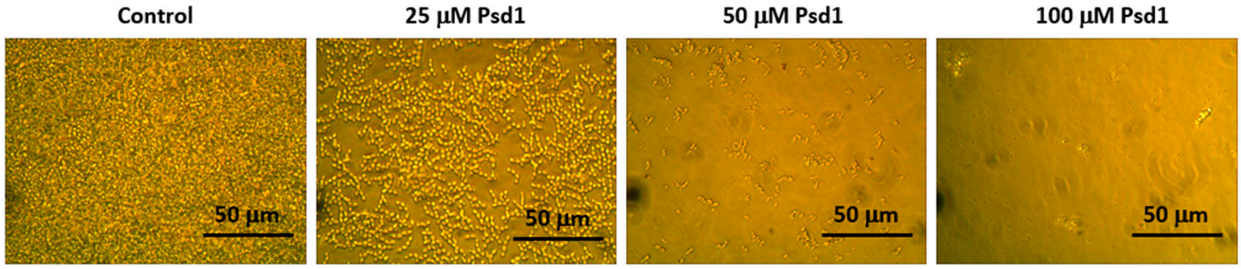

Figure S1. Antifungal activity of pea defensin Psd1 against clinical isolate of *C. albicans* v47a3 (×400 magnification).

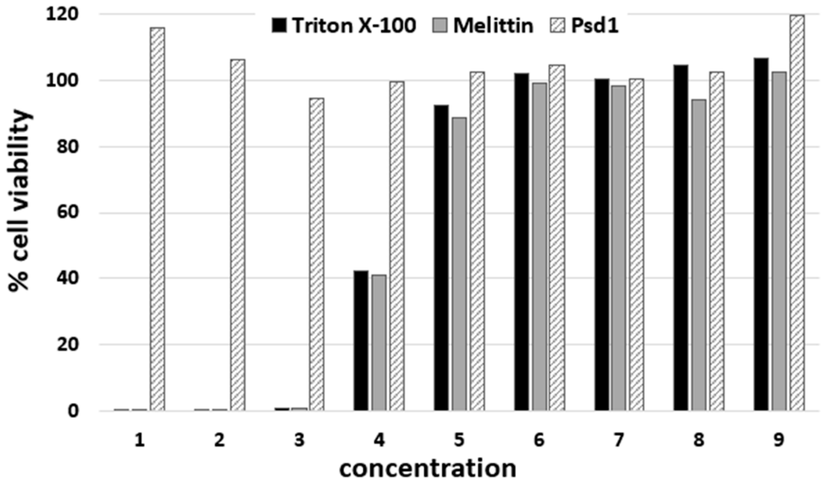

Figure S2. Cytotoxicity assay of different concentrations of pea Psd1, membrane-active peptide melittin from the venom of honeybees and Triton X-100. PBMCs were incubated 24 h with two-fold dilutions of compounds and next 16 h after resazurin adding. The concentrations used (1-9): Psd1 or melittin – 50, 25, 12.5, 6.25, 3.125, 1.56, 0.78, 0.39, 0.19 µM; Tritone X-100 – 0.1, 0.05, 0.025, 0.0125, 0.00625, 0.003125, 0.00156, 0.00078, 0.00039%.

Table S2. Phenotypic characteristics of clinical isolate of *Candida albicans* v47a3.

Characteristic	Substance
Hydrolysis	Glucose<br>Maltose<br>Galactose<br>ϱ-Nitrophenyl-N-acetyl- β,D-galactosaminide<br>ϱ-Nitrophenyl-α,D-glucoside<br>Proline-β-naphthylamide<br>Histidine β-naphthylamide
No hydrolysis	Sucrose<br>Trehalose<br>Raffinose<br>Fatty acid ester<br>ϱ-Nitrophenyl-β,D-glucoside

	σ-Nitrophenyl-β,D-galactoside<br>ϱ-Nitrophenyl-α,D-galactoside<br>ϱ-Nitrophenyl-β,D-fucoside<br>ϱ-Nitrophenyl phosphate,<br>ϱ-Nitrophenyl phosphoryl- choline<br>Urea<br>Proline-β-naphthylamide<br>Leucyl-glycine β-naphthylamide

Table S3. Sensitivity of the clinical isolate of *Candida albicans* v47a3 to conventional antimycotics (according to [31]).

Antifungal agent	MICs (mg/L)
Amphotericin B	0.5
Anidulafungin	0.03
Fluconazole	8
Flucytosine	≤0.06
Itraconazole	0.12
Micafungin	0.008
Posaconazole	0.12
Voriconazole	0.5
Caspofungin	0.06

MIC – minimal inhibitory concentration (>90% inhibition of fungal growth for amphotericin B, but 50% growth inhibition for other compounds).

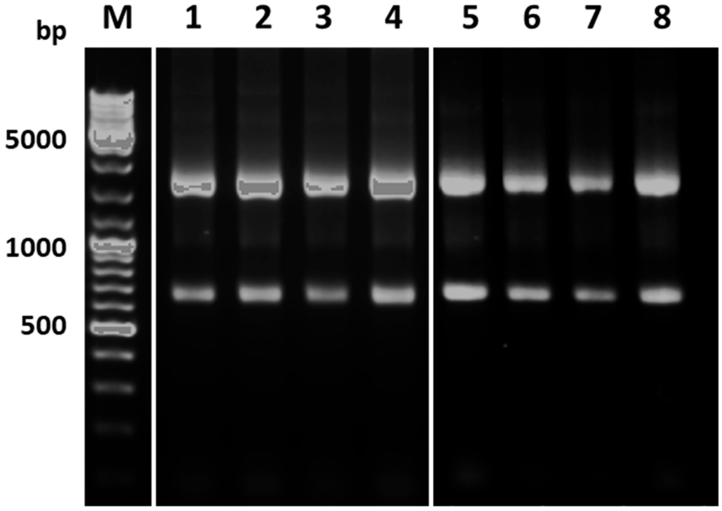

Figure S3. Caco-2 RNA quality as shown by 1.5% agarose electrophoresis. M – DNA ladder of varying length; 1-4 and 5-8 – Caco-2 RNA obtained after incubation during 4 and 24 h, correspondently. 1,5 – RNA from control Caco-2 cells; 2,6 – RNA from Caco-2 cells incubated with 2 μM pea Psd1; 3,6 – RNA from Caco-2 cells incubated with *C. albicans* in 5 × 10^4 cells/ml; 4,7 – RNA from Caco-2 cells incubated with *C. albicans* and pea defensin in the same concentrations.

Table S4. List of synthetic oligonucleotide primers used for study of epithelial cell responses.

Gene	Gene Bank	Forward	Reverse	Amplicon Size	Reference No.
GAPDH	NM_02046	GGGGAGCCAAAAGGGTCATCATCT	GAGGGGCCATCCACAGTCTTCT	235 bp	[34]
HBD-2	NM_004942	ATCTCCTCTTCTCGTTCCTC	ACCTTCTAGGGCAAAAGACT	126 bp	[35]
IL-1β	NM_000576.3	CGATGCACCTGTACGATCAC	CAGCTGTAGAGTGGGCTTATC	250 bp	-
IL-8/CXCL8	NM_000584.4	CTTGGCAGCCTTCCTGATTT	AACTTCTCCACAACCCTCTG	249 bp	-

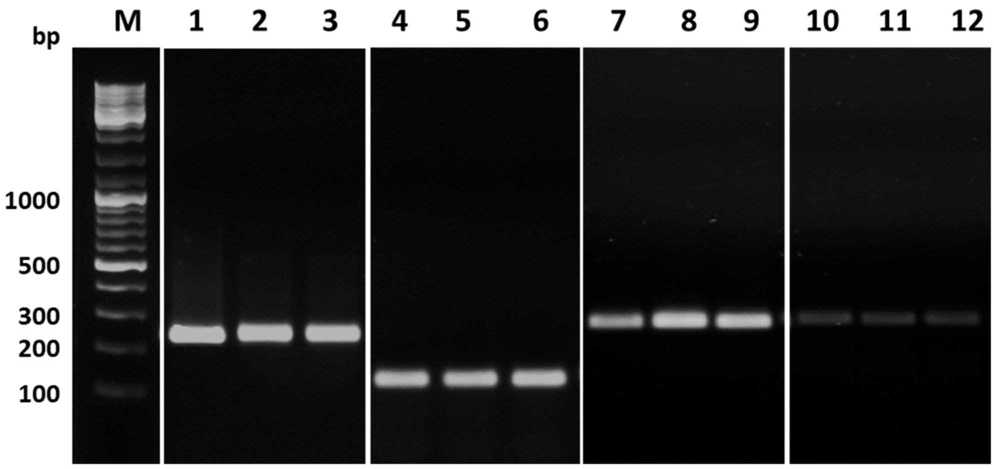

Figure S4. Agarose gel electrophoresis (1.5%) of real-time PCR products amplified using specific primer pair on *GAPDH* (1-3), *HBD-2* (4-6), *IL-1β* (7-9) and *IL-8/CXCL8* (10-12) genes. M – DNA ladder of varying length.
